# Isolation and structure elucidation of the compounds from *Teucrium hyrcanicum* L. and the investigation of cytotoxicity, antioxidant activity, and protective effect on hydrogen peroxide-induced oxidative stress

**DOI:** 10.1186/s12906-023-04262-8

**Published:** 2023-12-12

**Authors:** Saeed Ghasemi, Mehdi Evazalipour, Nastaran Peyghanbari, Ehsan Zamani, Peter Bellstedt, Mahan Molaee, Diba Eghbali Koohi, Fatemeh Yousefbeyk

**Affiliations:** 1 Department of Medicinal Chemistry, School of Pharmacy, Guilan University of Medical Sciences, Rasht, Iran; 2 Department of Pharmaceutical Biotechnology, School of Pharmacy, Guilan University of Medical Sciences, Rasht, Iran; 3 Department of Pharmacognosy, School of Pharmacy, Guilan University of Medical Sciences, Rasht, Iran; 4 Department of Pharmacology and Toxicology, School of Pharmacy, Guilan University of Medical Sciences, Rasht, Iran; 5 Institute of Clinical Chemistry, University of Zurich & University Hospital Zurich, Zurich, Switzerland

**Keywords:** Phenolic compounds, Flavonoid, Acteoside, Quercetin, Antioxidant, Cytotoxicity, Oxidative stress

## Abstract

**Background:**

*Teucrium hyrcanicum* L. (family Lamiaceae) is widely distributed in the North and Northwest of Iran. It has been used in the form of tea, tonic, and tincture for the treatment of various diseases such as cough, rheumatism, and fever.

**Methods:**

In this study, the total phenolic and flavonoid contents, antioxidant and cytotoxic activities of methanol extract and different fractions of *T. hyrcanicum* were measured. Furthermore, the potential ability of *T. hyrcanicum* to protect against H_2_O_2_-induced oxidative stress was tested on the NIH3T3 cell line. Then, the isolation and structure elucidation of the compounds were performed on the most potent fractions. Finally, the quantification of isolated compounds in methanol extract (ME) was done by the HPLC method. Isolated phytochemicals were assessed for the cytotoxic and antioxidant activities.

**Results:**

The results indicated that the methanol fraction (MF) had the highest amount of phenolic and flavonoid contents (69.36 mg GAE/g extract and 68.95 mg QE/g extract). The highest radical scavenging activities were observed from MF and ME (IC_50_ 44.32 and 61.12 μg.ml^−1^, respectively). The best cytotoxicity was obtained by ethyl acetate fraction (EF) against A431 and MCF7 cell lines (IC_50_ values of 235.4and 326.6 μg.ml^−1^, respectively). The pretreatment with MF exerts the highest reduction in malondialdehyde (MDA) formation (IC_50_ 2.51 μM, *p* < 0.001) compared to the H_2_O_2_ group (5.77 μM). Also, MF significantly inhibited H_2_O_2_-induced Glutathione (GSH) oxidation (*p* < 0.001). Furthermore, two phenolic compounds, acteoside and quercetin, were isolated and identified in MF and EF, respectively. The IC_50_ values of acteoside and quercetin in the DPPH assay were 7.19 and 5.56 µg.ml^−1^, respectively. Both quercetin and acteoside significantly reduced the MDA formation and inhibited GSH oxidation, which was comparable with BHA (as a standard antioxidant) (*p* < 0.05). Acteoside demonstrated significant cytotoxicity against all tested cell lines (IC_50_ = 32 to 145 μg.ml^−1^). The HPLC quantification of isolated compounds revealed that the quantity of acteoside and quercetin in ME were 93.31 and 16.87 μg.mg^−1^, respectively.

**Conclusion:**

The isolated compounds (quercetin and acteoside) had significant antioxidant activities and revealed a protective effect on H_2_O_2_-induced oxidative stress which was comparable with BHA.

**Supplementary Information:**

The online version contains supplementary material available at 10.1186/s12906-023-04262-8.

## Background

Oxidative stress is one of the main reason for free radical formation in food, drugs, and living organisms [1, 2]. It was demonstrated that exposure to free radicals such as superoxide anion radical (O•_2_^−^), hydroxyl radical (•OH), and peroxyl radical (ROO•) can lead to lipid peroxidation, protein denaturation, DNA oxidation, strand breakage, and platelet dysfunction [1–4]. These destructive effects can potentially lead to cancer, cardiovascular abnormalities, Alzheimer’s disease, fibrosis, kidney and liver disease, arthritis, atherosclerosis, neurodegenerative disorders, and aging [1]. Also, lipid peroxidation is the primary cause of food deterioration and production of unwanted compounds during processing and storage [5]. The natural antioxidants, found in medicinal and dietary plants, fruits, and vegetables, can prevent oxidative damage and provide health benefits by scavenging reactive free radicals [2]. Various bioactive phytochemicals, such as vitamins, polyphenols, terpenoids, polysaccharides, and alkaloids, have been proven to have potential antioxidant effects [6–10]. The growing interest in using natural antioxidants was even more highlighted when researchers indicated that the most commonly used synthetic antioxidants like butylated hydroxyanisole (BHA) and butylated hydroxytoluene (BHT) were suspected to have genotoxic and carcinogenic effects [1, 2]. Phenolic phytochemicals (e.g., phenolic acids, flavonoids, quinones, coumarins, lignans, stilbenes, and tannins) can inhibit the oxidative process by scavenging free radicals and reacting oxygen species (ROS). The mechanism of this action is related to their aromatic structures and highly conjugated system with several hydroxyl groups, which can act as good electron or hydrogen atom donors [11]. There are pieces of evidence that indicate phenolic compounds such as flavonoids (as free radical scavengers) can exert antitumor activities by inducing apoptosis as well as inhibiting invasion and metastasis in various cancer cell lines [12, 13].

The genus *Teucrium* (family Lamiaceae) is a widespread genus with about 340 different species [14–16]. Many of them are well-known for valuable therapeutic properties and have been used in the form of tea, tonic, and tincture for the treatment of diseases such as cough, rheumatism, diarrhea, fever, diabetes, antiseptic, anthelmintic, and flatulence [14, 15, 17]. So far, different phytochemicals have been reported from species of this genus, including monoterpenes, polyphenols, flavonoids (such as apigenin and rutin), sesquiterpenes, fatty acids, steroids (such as β-sitosterol and stigmasterol), and iridoids [14, 18]. Moreover, numerous biological activities like antimicrobial, antioxidant, antipyretic, anti-inflammatory, antiulcer, antimalarial, anti-allergic, anthelmintic, and cytotoxic activities have been demonstrated [14]. This genus comprises 12 species in the flora of Iran [19], and among them, *Teucrium hyrcanicum* L. is wildly distributed in the North and Northwest of the country. Until now, anti-inflammatory, anti-nociceptive, antibacterial, and anti-acetylcholine esterase activities of *T. hyrcanicum* have been reported [20, 21]. The investigation of the essential oil of this species revealed the presence of (E)-β-farnesene, hexahydrofarnesyl acetone, linalool, dihydroedulane, and ar-curcumene, as the major compounds [21]. Besides, flavonoids (pedalitin and luteolin 7-O-B-D-glucopyranoside), and diterpenes furolactones have been isolated from *T. hyrcanicum* [22, 23].

To find natural antioxidants from edible plants, in this study, the antioxidant activities of methanol extract and different fractions of *T. hyrcanicum* were measured by DPPH radical scavenging assay and phosphomolybdenum reduction test. Furthermore, the potential ability of *T. hyrcanicum* for protection against H_2_O_2_-induced oxidative stress was tested on NIH 3T3 cell line. Also, the total phenolic and flavonoid contents were measured. Likewise, the cytotoxicity was tested against three cancerous cell lines, including A431 (epidermoid carcinoma), MCF7 (breast cancer), HepG2 (liver hepatocellular carcinoma), as well as HU02 (Foreskin fibroblast) as a normal cell line using MTT assay. Also, the isolation and structural elucidation of compounds were reported from the fractions with the highest biological activities. The quantitative HPLC analysis of methanol extract was performed. Finally, two pure compounds were tested for their antioxidant and cytotoxic activities.

## Methods

### Reagents and chemicals

Gallic acid, quercetin, 2,2-Diphenyl-1-picrylhydrazyl (DPPH•), and BHA (Butylated hydroxyanisole) were obtained from Sigma chemical Company (USA). Aluminum trichloride (AlCl_3_), Folin–Ciocalteu’s phenol reagent, ammonium molybdate tetrahydrate, thiobarbituric acid, dithionitrobenzoic acid, and sodium bicarbonate were purchased from Merck, Germany. Dulbecco's Modified Eagle Medium (DMEM) and fetal bovine serum were obtained from Gibco and Invitrogen, respectively. HPLC-grade acetonitrile and acetic acid were purchased from DUKSAN, Korea. The three cancerous cell lines (including MCF7, HepG2, and A431) were purchased from the Iranian Biological Resource Center. NIH3T3 and Hu02 cell lines were purchased from the National Cell Bank of Pasteur Institute (Tehran, Iran). Other used reagents and solvents were of analytical grade.

### General procedures

Column chromatography was performed on Sephadex LH-20 (Fluka, Switzerland). Silica gel 60 F254 precoated plates (Merck, Germany) were used for TLC. HPLC separations were conducted on a Knauer system (Smart line system, Germany), Eurospher column (250 × 20 mm i.d.) and a PDA detector (λ: 210–400 nm). HPLC quantitative analysis was performed on a Waters Alliance e2695 HPLC apparatus equipped with Waters 2998 photodiode array (PDA) detector, and C18 reversed-phase column (Waters spherisorb, S5 ODS2, 4×250 mm, 5 μm). Data acquisition and quantitation were performed by Empower 3 software (Waters, USA). ^1^H and ^13^ C-NMR spectra were acquired using a Bruker NMR spectrometer (500 MHz for ^1^H and 125 MHz for ^13^C) with tetramethylsilane as an internal standard, and chemical shifts are given in δ (ppm). FT-IR spectra were obtained with a Spectrum Two FT-IR spectrometer with UATR accessory (PerkinElmer, USA) in the range of 400–4000 cm.^−1^

### Plant material, extraction, and fractionation

Aerial parts of the plant were collected from Rostam Abad mountain, Guilan province, in July 2020. The plant collection complies with relevant guidelines and regulations of plant protection at Guilan University of Medical Sciences (GUMS). The plant was identified, and the scientific name was confirmed by Dr. Fatemeh Yousefbeyk, Associate Professor of Pharmacognosy, School of Pharmacy, GUMS. The voucher specimen (114-HGUM) was deposited in the herbarium of the School of Pharmacy, GUMS, where it is publicly available. The aerial parts of *T. hyrcanicum* (500 g) were dried at room temperature, powdered, and extracted with methanol by percolation method (7 days). The methanol extract of *T. hyrcanicum* was dried by a rotary vacuum evaporator (Heidolph, Germany) to give 92 g dried extract. A portion of 90 g of dried methanol extract was subjected to the fractionation using the liquid–liquid method [24]. The solvents, including hexane, ethyl acetate, and methanol, were added in the order of increasing polarity. First, 200 ml of hexane was added to the methanol extract and mixed using an ultrasonic bath (Elma, Germany) for 15 min. The supernatant was collected, and the process was repeated five times. Then, ethyl acetate and methanol solvents (200 ml × 5, each) were added successively, and the same procedure was repeated. Finally, all the fractions were dried using a rotary evaporator to obtain 10.68, 6.54, and 63.72 g of each fraction, respectively. The concentrated fractions were kept at 4°C for further investigation.

### Isolation of compounds

Isolation of compounds was carried out on the most potent fractions, including ethyl acetate fraction (EF) and methanol fraction (MF). First, the methanol fraction (50 g) was dissolved in distilled water and extracted by n-BuOH (10,100× ml). The butanol fraction (6.5 g) was fractionated using a Sephadex LH20 column using MeOH as an eluent (5×30 cm, ֺflow rate 0.5 ml.min^−1^) to get 13 fractions (M1-M13). Fraction M2 (500 mg) was submitted to reversed phase semi-preparative HPLC. The mobile phase consisted of H_2_O/acetic acid (99:1) (solvent A) and acetonitrile (solvent B). The initial mobile phase, including 9:1 (A/B), was delivered at a flow rate 5 ml.min^−1^ for 10 min, before it was changed to 7:3 (A/B) until 20 min, and remained in this ratio for 10 min (30 min after starting point). Then, chromatography was carried on with the ratio 1:1 (A/B) for another 10 min (40 min after the starting point). Finally, a gradient elution was used to reach 100% B in 20 min (60 min after the starting point). It afforded compound 1 (Rt: 22.4 min). The separation elution curve is provided in Supplementary file.

The ethyl acetate fraction (5 g) was subjected to a Sephadex LH20 column with MeOH as an eluent (5×30 cm, flow rate 0.5 ml.min^−1^) and 20 fractions were collected (E1-E20). The sub-fraction E4 (170 mg) was loaded on a Sephadex LH20 column (2×40 cm, flow rate 0.5 ml.min^−1^) with MeOH for further isolation and the compound 2 was isolated.

### Measurement of total phenolic content

The Folin-Ciocalteu colorimetric method was carried out to determine the concentration of total phenolics in methanol extract and fractions of this plant [25]. To begin, methanol extract and fractions (1 ml, each) were separately mixed with Folin-Ciocalteu reagent (5 ml, diluted tenfold with distilled water). The mixtures were allowed to stand for 10 min. A solution of sodium bicarbonate at concentration of 75 g.l^−1^ was also prepared and 4 ml was added to each sample. After 30 min in darkness, the absorbance was read by UV–Vis spectrophotometer at 765 nm. Different dilutions of gallic acid (GA), including 20, 50, 75, 100, and 150 µg.ml^−1^ were prepared for plotting the calibration curve. All the tests were repeated three times. Total phenolic contents were expressed as mg of gallic acid equivalents (GAE)/g extract [26].

### Measurement of total flavonoid content

The concentration of the total flavonoids was determined using a method described by Saeidnia and Gohari [27, 28]. First, 5 ml of each sample was added to 5 ml of aluminum trichloride (AlCl_3_) (2% in methanol). After 10 min, the absorbance of the mixture was measured at 415 nm using a UV–Vis spectrophotometer. A standard curve was plotted for measuring the total flavonoid content using different concentrations of quercetin as the standard (10, 25, 50, 75, and 100 µg.ml^−1^). The tests were repeated three times for each sample. Finally, total flavonoid content was expressed as mg of quercetin as equivalents (QE)/g extract [26, 29].

### In-vitro antioxidant activity

#### DPPH radical scavenging activity

The methanol extract, fractions, and isolated compounds were tested for their antioxidant activities using the 2,2'-diphenyl-1-picrylhydrazyl (DPPH) radical scavenging assay. Briefly, 2 ml of DPPH solution (40 μg.ml^−1^) was added to 1 ml of each sample with different concentrations. The samples were kept at room temperature for 30 min before the absorbance was measured at 517 nm. The tests were repeated three times for each concentration. BHA was used as the positive control. The control comprised samples (1 ml) and water (2 ml). The blank contained distilled water (1 ml) and DPPH solution (2 ml). The inhibition activity was determined by the following formula [30]:

$$\%Inhibition=100-\left[\frac{(\mathrm{As}-\mathrm{Ac})}{\mathrm{Ab}}\right]\times 100$$

Where A_s_ was the absorbance of sample, A_c_ was the absorbance of control, and A_b_ was the absorbance of blank. The IC_50_ values were expressed as the concentration of each sample (µg.ml^−1^) resulting in 50% inhibition and calculated from the graph-plotted of inhibition (%) against samples concentration.

### Determination of total antioxidant capacity by phosphomolybdenum reduction assay (PRA)

The antioxidant capacity of the methanol extract and fractions were determined by the phosphomolybdenum reduction assay (PRA) [31]. Generally, the samples reduce Mo(VI) to Mo(V), forming specific green phosphate-Mo(V) compounds in acidic pH. Briefly, 0.3 ml of each sample was mixed with 3 ml of a reagent mixture (0.6 M sulfuric acid, 28 mM sodium phosphate, and 4 mM ammonium molybdate) in a test tube. All samples were incubated in an oil bath at 95°C for 90 min. After cooling to room temperature, the absorbance of each mixture was measured at 695 nm [31]. The total antioxidant capacities are expressed as mg of α-tocopherol equivalents (αTE/g extract), using the following linear equation plotted by α-tocopherol as the standard: Y = 0.0024X + 0.004; *R*^*2*^ = 0.998. In this equation, Y is the absorbance at 695 nm, and X is the concentration of αTE (μg.ml^−1^).

### Cellular antioxidant activity

#### Cell culture and treatment

NIH3T3 fibroblast cells were cultured in DMEM medium containing 10% fetal bovine serum (FBS), supplemented with 100 U.ml^−1^ penicillin and streptomycin, and 2 mM L-glutamine in a humidified atmosphere of 95% air, 5% CO_2_ at 37 °C [32, 33]. The cell counts and viability were determined by trypan blue staining and a density of 1 × 10^6^ cells/well were seeded into 6-well cell culture plates. The cells were treated with methanol extract (ME), methanol fraction (MF), ethyl acetate fraction (EF), two isolated compounds, including quercetin and acteoside, and BHA as the standard antioxidant (at concentrations equal to IC_50_ of DPPH assay), for 24 h. Then, cells were exposed to H_2_O_2_ (100 µM) for 2 h [34]. Finally, cell suspensions were homogenized and used for other experiments.

#### Measurement of lipid peroxidation

For the evaluation of lipid peroxidation, the end product of the reaction, malondialdehyde (MDA), was assessed using thiobarbituric acid (TBA) as the indicator [32, 33]. Briefly, 0.25 ml phosphoric acid (0.05 M) was added to 0.2 ml of cell homogenates with the addition of 0.3 ml TBA (0.2%). Later, the samples were placed in a boiling water bath for 30 min. Next, the tubes were transfered to an ice bath and n-butanol was added to each tube. Then, they were centrifuged at 3500 rpm for 10 min. The amount of malondialdehyde (MDA) formed in each sample was assessed by measuring the absorbance of the supernatant at 532 nm with an ELISA reader (Epoch™ Microplate Spectrophotometer, BioTek, USA).

#### Measurement of glutathione (GSH) concentration

GSH concentration of the cell homogenates was determined by dithionitro benzoic acid (DTNB) [35]. The method is based on the formation of yellow color when DTNB reacts with compounds containing sulfhydryl groups. To begin the reaction, 2.3 ml of potassium phosphate buffer (0.2 M, pH 7.6) was added to 0.2 ml of cell lysate supernatant. Then, 0.5 ml of DTNB (0.001 M) was added to the solution. The absorbance of the products was observed after 5 min at 412 nm.

### Cell cultures and cytotoxicity assay

The methanol extract, fractions, and isolated compounds were tested for cytotoxic activities against three cancerous cell lines (including MCF-7, HepG2, and A431) and the normal cell line (Hu02) by methyl thiazol tetrazolium (MTT) assay. Briefly, for each cell line, 10^4^ cells per well were seeded in a 96-well plate in complete DMEM and incubated for 24 h at 37°C in a humidified atmosphere containing 5% CO_2_. Next, non-adherent cells were removed, and adherent cells were treated with different concentrations of samples (62.5, 125, 250, 500, and 1000 μg.ml^−1^). The treated cell lines were incubated in the same condition for 48 h. Afterward, the MTT solution (20 µl, 5 mg.ml^−1^ in PBS) and DMEM (180 µl) were added to seeded cells and incubated for 4 h. Then, the supernatants were removed, DMSO (150 µl) was added to each well, and the samples were shaken for 10 min to dissolve formazan crystals [36, 37]. The amount of purple formazan produced is related to the number of viable cells [38]. The solution optical density (OD) was measured by an absorbance microplate reader (BioTek) at 490 nm, using a reference wavelength of 630 nm. Each MTT assay was repeated three times. The percentage of viable cells in each treated group was calculated based on the following equation:

$$\mathrm{\%\ Viability}=(\frac{\left[\mathrm{OD\ treated\ group}-\mathrm{OD\ background}\right]}{\left[\mathrm{OD\ control}-\mathrm{OD\ background}\right]})\times 100$$

IC_50_ of samples against each cell line was defined as the concentration in which only 50% of cells were alive and computed using GraphPad Prism (Version 8, GraphPad Software, USA) [39, 40].

### Quantitative HPLC analysis of *T. hyrcanicum* methanol extract

The quantitative analysis of acteoside and quercetin, as major compounds, was performed on the methanol extract of *T. hyrcanicum* using a Waters Alliance e2695 HPLC apparatus equipped with the Waters 2998 photodiode array (PDA) detector and C18 reversed-phase column (Waters spherisorb, S5 ODS2, 4×250 mm, 5 μm). The absorbance changes were monitored at 210 nm to 400 nm. Data acquisition and quantitation were performed using Empower 3 software (Waters, USA). The mobile phase consisted of H_2_O/acetic acid (99:1) (solvent A) and acetonitrile (solvent B). The flow rate of 1 ml.min^−1^ was used for a total run time of 60 min. Elution was performed using the following gradient program: 1–10 min: 10% B, 10–20 min: linear gradient from 10 to 30% B, 20–60 min: linear gradient from 30 to 100% B. The peak related to each compound was identified by comparison of DAD absorbance spectra with external standards. Acteoside and quercetin, as standard compounds, were used to prepare two different concentration levels (100 to 1000 μg.ml^−1^). The peak areas of each standard were plotted against the corresponding standard concentrations (μg.ml^−1^) using linear regression to create standard curves. The determination coefficient (R^2^) was calculated by means of the least-square analysis. Limits of detections (LODs) and limits of quantitations (LOQs) were determined using the expressions 3.3σ/s and 10σ/s, respectively, in which σ is the intercept standard deviation, and s is the slope of the calibration curve.

### Statistical analysis

All the results were expressed as the mean ± SD (standard deviation of the mean). The statistical analyses were done using the SPSS software, version 13 (SPSS, Chicago, IL). All the statistical significance was calculated using the one-way analysis of variance (ANOVA) test, followed by the post-hoc Tukey test. Statistical significance was set at *p* < 0.05.

## Results and discussion

### Total phenolic and flavonoid contents

The total phenolic content of methanol extract and fractions were measured by the Folin-Ciocalteu method. As is presented in Table 1, the highest content of phenolic compounds was reported from MF (69.36 ± 0.02 mg of GAE/g extract), followed by ME (58.13 ± 0.02 mg of GAE/g extract), using the standard curve of gallic acid (y = 0.00096x − 0.0365, *R*^*2*^ = 0.995). Moreover, the total flavonoid content was measured, by standard curve of flavonoid quercetin (y = 0.0187x—0.0254, *R*^*2*^ = 0.975) and reported in the range of 68.95 ± 0.05 to 18.48 ± 0.01 mg QE/g extract. The MF and ME had the highest amount of flavonoids (68.95 ± 0.05 and 53.53 ± 0.01 mg of QE/g extract).

**Table 1:** Total phenolic and flavonoid contents of methanol extract and different fractions of *T. hyrcanicum*

sample	Total phenolic content^b^	Total flavonoid content^c^
ME^a^	58.13 ± 0.02	53.53 ± 0.01
HF	33.94 ± 0.01	18.48 ± 0.01
EF	36.02 ± 0.07	25.43 ± 0.03
MF	69.36 ± 0.02	68.95 ± 0.05

^a^*ME* methanol extract, *HF* hexane fraction, *EF* ethyl acetate fraction, *MF* methanol fraction, of *T. hyrcanicum*

^b^mg of GAE/g extract

^c^mg of QE/g extract; the results are expressed as mean ± standard deviation of three independent experiments

A literature survey showed that the total phenolic content of different species of *Teucrium* has been reported. In Table 2, the reported amounts of total phenols in methanol extract of different *Teucrium* species were summarized. As is presented, the phenolic content of *T. hyrcanicum* is in the range of previous studies.

**Table 2:** The comparison of total phenolic content of methanol extract of *T. hyrcanicum* with other *Teucrium* species

	Name of species	Total phenolic content^a^	Ref
1	*T. chamaedrys*	243.65 ± 3.46	[41]
2	*T. arduini*	200.35 ± 0.46	[17]
3	*T. scordium* subsp*. scordioides*	186.02 ± 0.91	[42]
4	*T. scordium* subsp*. scordium*	178.20 ± 1.11	[42]
5	*T. chamedrys*	172.50 ± 1.26	[42]
6	*T. flavum*	171.08 ± 0.38	[17]
7	*T. chamaedrys*	169.50 ± 0.22	[43]
8	*T. polium*	124.62 ± 1.05	[17]
9	*T. stocksianum*	115.55 ± 0.74	[44]
10	*T. hyrcanicum*	58.13 ± 0.02	Present study
11	*T. botrys*	56.62 ± 0.99	[45]
12	*T. montanum*	45.41 ± 0.85	[1] 7 g in
13	*T. trifidum*	14.33 ± 0.087	[46] 0

^a^mg of GAE/g extract

### Isolation and purification of compounds

**Fig. 1:**
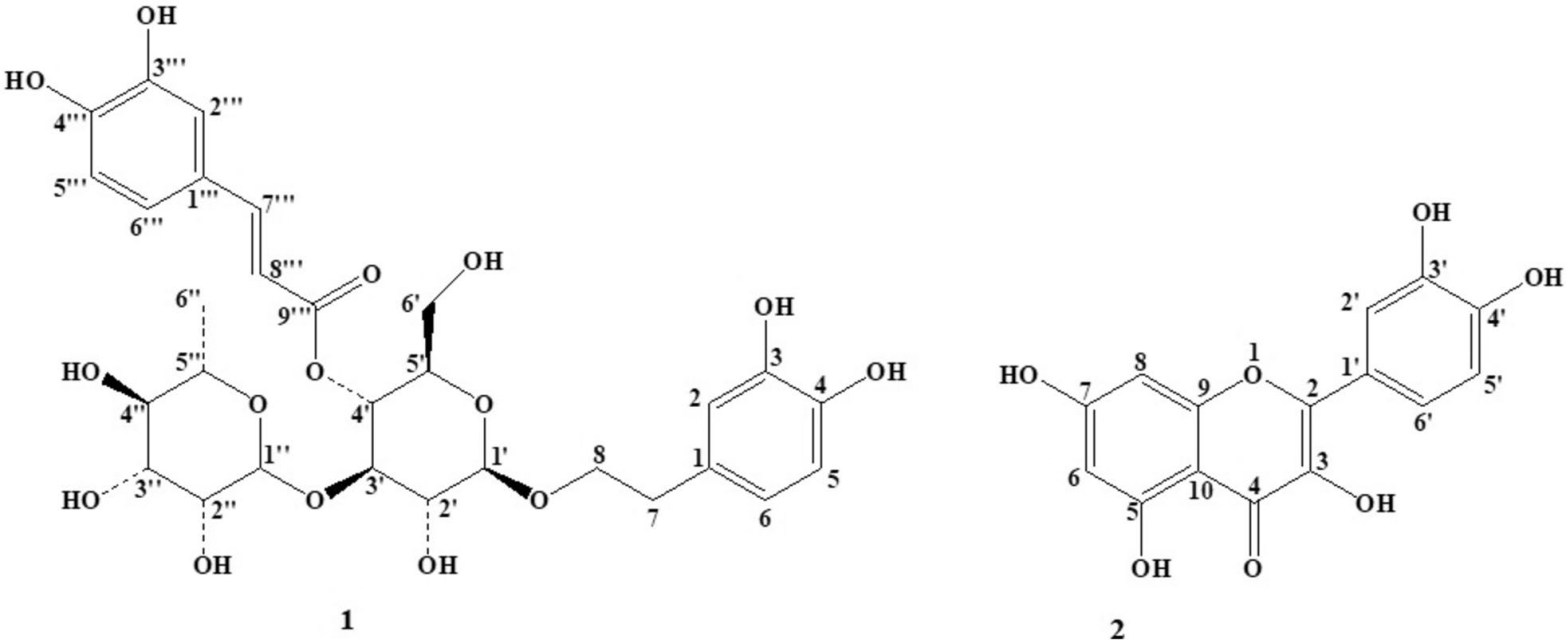
Structures of acteoside (1) and quercetin (2), isolated from *T. hyrcanicum*

The methanol and ethyl acetate fractions were subjected to phytochemical investigation to purify the main bioactive compounds. For the first time, acteoside and quercetin were isolated, and identified in MF and EF of *T. hyrcanicum,* respectively (Fig. 1).

The identification of compounds was performed by comparison of their spectral data (FT-IR, ^1^ H and ^13^C-NMR) with those published in the literature [47, 48]. The peak assignments of the detected compounds are reported as follows:

Compound 1: FT-IR (νmax): 3290 (OH), 1659 (C = O), 1605 (Alkene C = C), 1442 and 1558 (aromatic C = C), 1158 (C–O–C); ^1^H NMR (DMSO-*d6*, 500 MHz): *δ* ppm 7.47(d, 1H, J = 15.5 Hz), 7.04 (s, 1H), 6.99 (d, 1H, J = 7.5 Hz), 6.77 (d, 1H, J = 7 Hz), 6.64 (s, 1H), 6.64 (s, 1H), 6.51 (d, 1H, J = 7.5 Hz), 6.21 (d, 1H, J = 16 Hz), 5.04 (s, 1H), 4.36 (d, 1H, J = 8 Hz), 3.6 (dd, 2H, J = 9 Hz, J = 15.75 Hz), 2.7 (t, 2H, J = 7.5 Hz), 0.97 (d, 3H, J = 6 Hz). ^13^C NMR (DMSO-*d6*, 125 MHz) *δ* ppm 166.18 (C9'''), 148.98 (C4'''), 146.05 (C3'''), 145.46 (C3), 145.46 (C7'''), 144.01 (C4), 129.60 (C1), 125.97 (C1'''), 121.92 (C6'''), 120.02 (C6), 116.78 (C2), 116.27 (C5'''), 115.94 (C5), 115.15 (C2'''), 114.02 (C8'''), 102.74 (C1'),101.69 (C1''), 79.56 (C3'), 74.98 (C2'), 74.96 (C5'), 72.13 (C4''), 70.99 (C2''), 70.85 (C3''), 70.73 (C8), 69.59 (C4'), 69.21(C5''), 61.20 (C6'), 35.66 (C7), 18.62 (C6'').

Compound 2: FT-IR (νmax): 3298 (OH), 1688 (C = O), 1606 (Alkene C = C), 1439 and 1576 (aromatic C = C), 1038 and 1266 (phenyl alkyl C–O–C); ^1^H NMR (DMSO-*d6*, 500 MHz): *δ* ppm 12.5 (s, 1H), 10.70 (brs, 1H), 9.34 (brs, 3H), 7.69 (s, 1H), 7.55 (d, 1H, J = 8Hz), 6.9 (d, 1H, J = 8Hz), 6.41 (s, 1H), 6.19 (s, 1H). ^13^C NMR (DMSO-*d6*, 125 MHz) *δ* ppm 176.3 (C4), 164.35 (C7), 161.19 (C9), 156.60 (C5), 148.16 (C2), 147.26 (C4´), 145.51 (C3´), 136.20 (C3), 122.42 (C6´ and C1´), 116.10 (C5´), 115.54 (C2´), 115.52 (C10), 98.64 (C6), 93.82 (C8).

Acteoside, also named verbascoside, is a phenylethanoid glycoside, which is among the most prevalent disaccharide caffeoyl esters [49]. It comprises caffeic acid, 3′,4′-dihydroxyphenylethanol, glucose, and rhamnose [50]. It has been isolated from several plants species, such as *Verbascum* species, *Buddleja brasiliensis*, *Striga asiatica*, *Olea europea*, *Paulownia tomentosa* var*. tomentosa*, *Lippia javanica*, *Lantana camara, Plantago lanceolate,* and *Cistanche deserticola* [49, 51–53]. Biological activities, such as anti-inflammatory and regulation of cell apoptosis, have been reported from this compound [49, 54, 55]. This is the first report of acteoside from *T. hyrcanicum.* Previously, Sadeghi et al. reported the presence of phenylethanoid glycosides such as verbascoside and rabinosyl-verbascoside in *T. chamaedrys* and *Teucrium sub spinosum*, respectively [56]*.*

The flavonol quercetin (3,31,41,5,7-pentahydroxyflavone) is among the most abundant flavonoids [57]. It exists in numerous types of vegetables and fruits, such as onions, apples, tea, and grapes [57, 58]. Several pharmacological properties, including anti-obesity, anti-inflammatory, cardioprotective, antihypertensive, antidiabetic, anti-hypercholesterolemic, and anti-atherosclerotic activities, have been reported from this compound [59, 60].

### Antioxidant activity

The methanol extract, different fractions, and isolated compounds from *T. hyrcanicum* were investigated for antioxidant activities by the DPPH radical scavenging assay. The IC_50_ results of DPPH assay varied from 44.32 to 205.40 µg.ml^−1^ (Table 3). Also, the highest radical scavenging activities were found in MF and ME (IC_50_ of 44.32 and 62.12 µg.ml^−1^, respectively). The IC_50_ values of two isolated compounds, acteoside and quercetin, were 7.19 ± 0.8 and 5.56 ± 0.9 µg.ml^−1^, respectively. BHA, the standard antioxidant, showed the IC_50_ value of 7.81 µg.ml^−1^.

**Table 3:** The antioxidant activities of *T. hyrcanicum* and isolated compounds

sample	DPPH^b^	PRA
ME^a^	62.12 ± 3.75	354 ± 0.02
HF	205.4 ± 8.98	297 ± 0.25
EF	194.57 ± 11.45	363.6 ± 0.04
MF	44.32 ± 5.5	347.5 ± 0.01
Acteoside	7.19 ± 0.8	-
Quercetin	5.56 ± 0.9	-
BHA	7.81 ± 1.2	-

^a^*ME* methanol extract, *HF* hexane fraction, *EF* ethyl acetate fraction, *MF* methanol fraction

^b^DPPH radical scavenging activity expressed as IC_50_ values (µg.ml^−1^); *PRA* phosphomolybdenum reduction assay (mg αTE/g extract); Values of the results are expressed as mean ± standard deviation of three independent experiments

Furthermore, the total antioxidant capacity was determined by phosphomolybdate reduction assay (PRA) (Table 3). The results revealed that EF, ME, and MF had the relatively close reducing ability (363.6 ± 0.04, 354 ± 0.02, and 347.5 ± 0.01 mg αTE/g extract).

Until now, different *Teucrium* species have been investigated for their antioxidant activities. Sharififar et al. indicated that the antioxidant activities of different fractions prepared from *T. polium*, growing in Kerman, Iran, were between 20.1 µg.ml^−1^ (methanol fraction) and 85.4 µg.ml^−1^ (chloroform fraction) [2]. Stankovic et al. showed that the antioxidant activities of different extracts of *T. chamaerdys* L. var. *glanduliferum* varied from 341.08 µg.ml^−1^ (petroleum ether extract) to 29.46 µg.ml^−1^ (methanol extract) [43]. In another study, Stankovic et al. investigated the antioxidant activities of various extracts from *T. montanum* var. *nontanum* and obtained results ranged from 29.41 (water extract) to 1388.03 µg.ml^−1^ (petroleum ether extract) [1]. The results of a study by Mazhangara et al. revealed that the antioxidant activities of methanol and ethanol extracts from *T. trifidum* were 17 and 12 µg.ml^−1^, respectively [46]. Furthermore, Vlase et al. reported that the hydroalcoholic extract from *T. chamaedrys* had an IC_50_ of 26.70 µg.ml^−1^ [41]. Another study conducted by Mukarram Shah et al. displayed that methanol extract of *T. stocksianum* exerted high antioxidant activity (IC_50_ = 12.5 µg.ml^−1^) [44]. Also, the antioxidant activities of different extracts from *T. barbeyanum* were tested and the IC_50_ ranged from 5.39 to 77 µg.ml^−1^. The best antioxidant activity was reported from ethyl acetate fraction [61]. The results of the present study exhibited that two isolated compounds, quercetin and acteoside, had the highest antioxidant effects in the DPPH radical scavenging assay. Therefore, *T. hyrcanicum* can potentially be used as a source of these natural antioxidants.

### Effects of *T. hyrcanicum* methanol extract, fractions, and isolated compounds on lipid peroxidation induced by H_2_O_2_

**Fig. 2:**
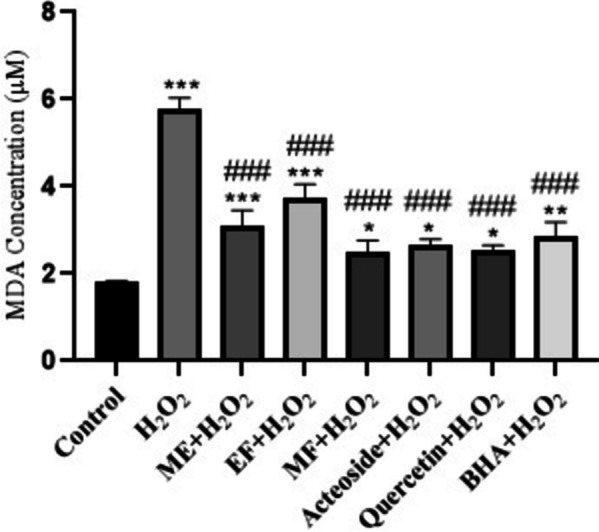
The effects of methanol extract (ME), methanol fraction (MF), ethyl acetate fraction (EF), acteoside, quercetin, and BHA on the malondialdehyde (MDA) level in NIH3T3 fibroblast cells treated with 100 mM H_2_O_2_ after 24 h. Values are the mean ± S.E.M. for six experiments; *: significantly different (*p* < 0.05) from the control; **: significantly different (*p* < 0.01) from the control; *** Significantly different (*p* < 0.001) from the control; ###: significantly different (*p* < 0.001) from H_2_O_2_ group

A significant increase (*p* < 0.001) in MDA concentration (5.77 μM) was observed in NIH3T3 fibroblast cells treated with 100 mM H_2_O_2_, after 24 h, compared to the control group (1.8 μM) (Fig. 2). Interestingly, pretreatment by *T. hyrcanicum* methanol extract, fractions, and isolated compounds significantly (*p* < 0.001) prevented MDA elevation in NIH3T3 fibroblast cells treated with H_2_O_2_, for 24 h. BHA, as a standard antioxidant, showed potential antioxidant activity by decreasing the MDA formation (2.86 μM, *p* < 0.01). Moreover, methanol fraction, quercetin, and acteoside exert the highest reduction in MDA formation (2.51, 2.55, and 2.66 μM, respectively), compared to the ME and EF.

Lipid peroxidation is associated with aging and various chronic health diseases, such as cancer and atherosclerosis (41). MDA is a crucial degradation product of lipid peroxidation and acts as an indicator for measuring the degree of lipid peroxidation (42). In this study, the MF, quercetin, and acteoside showed better protective activities than BHA. They showed significant antioxidant properties, which lead to a lower degree of lipid peroxidation and higher hydroxyl radical scavenging abilities.

### Effects of *T. hyrcanicum* methanol extract, fractions, and isolated compounds on GSH oxidation induced by H_2_O_2_

**Fig. 3:**
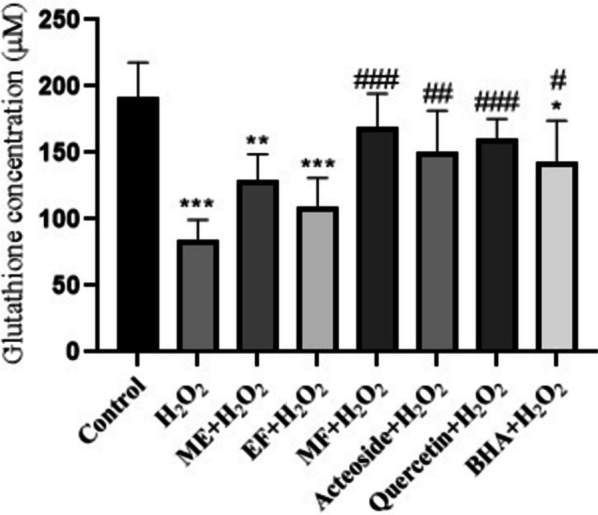
The effects of methanol extract (ME), methanol fraction (MF), ethylacetate fraction (EF), acteoside, quercetin, and BHA on GSH oxidation induced by H_2_O_2_ in NIH3T3 fibroblast cells treated with 100 mM H_2_O_2_, after 24 h. Values are the mean ± S.E.M. for six experiments; *: significantly different (*p* < 0.05) from the control; **: significantly different (*p* < 0.01) from the control; *** Significantly different (*p* < 0.001) from the control;# significantly different (*p* < 0.05) from H_2_O_2_ group; ## significantly different (*p* < 0.01) from H_2_O_2_ group; ###: significantly different (*p* < 0.001) from H_2_O_2_ group

As shown in Fig. 3, exposure to H_2_O_2_ leads to a significant decrease in GSH level compared to the control group (*p* < 0.001). BHA showed significant antioxidant activity by elevating GSH level (142.69 μM, *p* < 0.05). The results showed that pretreatment with MF, quercetin, and acteoside considerably inhibited the H_2_O_2_-induced GSH oxidation in NIH3T3 fibroblast cells (*p* < 0.001, *p* < 0.01, and *p* < 0.001). Pretreatment with ME and EF could not significantly increase the GSH level related to H_2_O_2_ group.

The GSH is a vital antioxidant defense agent against free radicals. GSH (ɣ-glutamylcysteinylglycine) is a ubiquitous low molecular weight thiol in eukaryotes and is the most abundant antioxidant within all cells. It acts to remove ROS and therefore, reduce the lifetime of the oxidative signals [62, 63]. Reactive oxygen species, such as superoxide anion, hydroxyl radical, and H_2_O_2_, are regularly produced intracellularly because of usual metabolic activities, and can cause cellular oxidative damage [64]. In this study, hydrogen peroxide was used to induce oxidative stress. It is one of the most frequently generated ROS. It can easily pass through a lipid membrane, reacting with metal ions to produce highly toxic hydroxyl radicals, resulting in oxidative stress. Hydrogen peroxide can deplete GSH levels in cells [65]. According to the results, the MF fraction of *T. hyrcanicum* can be considered a significant antioxidant, even more potent than BHA in increasing GSH level (169.69 μM).

### Cytotoxicity

The anti-proliferative activities of methanol extract, different fractions, and isolated compounds were evaluated on three cancerous cell lines, including the human breast adenocarcinoma (MCF-7), hepatocellular carcinoma (HepG2), and epidermoid carcinoma (A431) cell lines as well as a normal cell line (Hu02) by MTT assay and IC_50_s were demonstrated in Table 4. The cell lines were subjected to the increasing doses of each sample ranging from 62.5 to 1000 μg.ml^−1^ for 48 h, which reduced the viability of these cell lines.

**Table 4:** The cytotoxic activities of methanol extract, fractions, and isolated compounds on cancerous and normal cell lines by MTT assay

samples	Cell line			
	MCF7^b^	A431	HepG2	Hu02
ME^a^	392.5 ± 20^c^	> 500	> 500	> 500
HF	459.5 ± 17.1	> 500	> 500	> 500
EF	326.6 ± 36.6	235.4 ± 34.8	> 500	> 500
MF	> 500	> 500	> 500	> 500
Acteoside	32 ± 0.54	103 ± 1.65	194.6 ± 1.22	> 500
Quercetin	389.60 ± 16.6	132.32 ± 12.1	338.3 ± 36.3	> 500

^a^*ME* methanol extract, *HF* hexane fraction, *EF* ethyl acetate fraction, *MF* methanol fraction

^b^MCF 7 (breast ductal carcinoma), A431 (epidermoid carcinoma), HepG2 (hepatocellular carcinoma)

^c^IC_50_: required concentration of each sample to inhibit cell proliferation by 50% expressed as μg.ml^−1^; IC_50_ ± standard deviation (data from three experiments)

Among different fractions, the best cytotoxicity was achieved by EF against A431 and MCF7 (235.4 and 326.6 μg.ml^−1^, respectively). Also, ME had cytotoxic activity on MCF7 with IC_50_ of 392 μg.ml^−1^. Other fractions exhibited lower cytotoxicity on tested cell lines (> 500 μg.ml^−1^). The isolated compound, acteoside, demonstrated significant cytotoxicity against all tested cell lines (ranging from 32 to 194.6 μg.ml^−1^). Quercetin represented its best antiproliferative activity against A431 (132.32 μg.ml^−1^).

So far, the genus *Teucrium* has been introduced as a natural source of anticancer compounds [42]. A literature survey demonstrated that the extracts of *T. polium* potentiate the cytotoxic and proapoptotic effects of anticancer drugs like doxorubicin, vincristine, and vinblastine against breast, epidermoid, bladder, and oral cavity epidermal cell lines [66]. In another study, the cytotoxic activity of aqueous extract from *T. stocksianum* was evaluated on MCF7 cell lines, and the IC_50_ value was 199.99 µg.ml^−1^ [67]. The methanol extracts of *T. polium*, *T. chamaedrys*, *T. botrys*, and *T. montanum* showed cytotoxic activities against HCT-116 (human colon cancer cell line) with the IC_50_ of 253.39, 190.07, 183.15, and 75.73 µg.ml^−1^, respectively [42].

So far, the extracts or isolated compounds have been investigated for anti-tumor activities. These diverse and complicated structures can trigger anti-inflammatory, anti-tumor, and/or anti-metastatic reactions and reduce multidrug resistance [54, 68]. In our study, acteoside exhibited the best cytotoxic activity against MCF7, followed by A431 cell lines. In another study, acteoside showed antiproliferative effect against HL-60 (human promyelocytic leukemia cells) with IC_50_ of 30 μM [69]. Acteoside at dose of 10 µM meaningfully decreased the proliferation of gastric adenocarcinoma (AGS) cells by 19%, and it was inactive on the colorectal adenocarcinoma (HT-29) cell line [70]. Acteoside efficiently inhibits the growth of oral squamous cell carcinoma (OSCC) cells [71]. This compound showed cytotoxic activity on HCT-116 (colon adenocarcinoma cell line) with IC_50_ of 32.49 μg.ml^−1^ [72]. The IC_50_ values of this compound on HEP-2 (human larynx epidermoid carcinoma) and RD (human rhabdomyosarcoma) cells were 55.6 and 36.24μg.ml^−1^, respectively [73].

Former studies proved that polyphenol antioxidants can cause antiproliferative effects on specific cancer cells by acting as prooxidant agents [42, 74, 75]. The prooxidant activities of phenolic compounds are mediated by forming phenoxyl radicals that can react with oxygen to produce oxygen radical (O_2_^●^) and quinone-semiquinones complex. These compounds can cause DNA damage and apoptosis in normal and cancer cells [74, 75]. It is suggested that the significant antioxidant activity of acteoside can be due to its prooxidant activity.

Flavonoids have recently attracted a lot of attention due to their potential role in cancer prevention and cancer therapy. They can regulate cell proliferation and pathways leading to cancer [76]. Quercetin is an important flavonoid that exists in many plants. Quercetin has potential anticancer effect due to antiproliferative, growth factor suppression, and antioxidant activities [77]. In this study, quercetin showed its best cytotoxic activity on A431 cell lines (IC_50_ of 132.32 μg.ml^−1^).

### The quantitative HPLC analysis of quercetin and acteoside in methanol extract

**Fig. 4:**
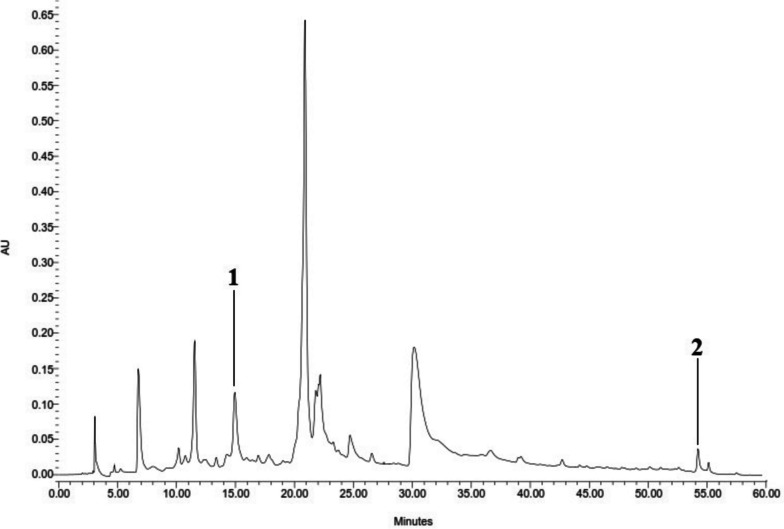
HPLC chromatogram of methanol extract from *T. hyrcanicum*, (1) acteoside (Rt 15 min), and (2) quercetin (Rt 54 min)

In this study, the quantitative analyses of acteoside and quercetin in methanol extract of *T. hyrcanicum* were performed by developing an HPLC method. Figure 4 depicted a typical HPLC chromatogram of *T. hyrcanicum* methanol extract*.* The acteoside and quercetin were detected at Rt 15 and 54 min, respectively. The quantities of these compounds in ME were 93.31 and 16.87 μg.mg^−1^ extract.

The results of method validation for analysis of quercetin and acteoside according to linearity, LOD, and LOQ revealed that the developed method was valid (Table 5). Outstanding linearity was obtained in the range of 10–1000 μg.ml^−1^, with *R*^*2*^ = 0.999 and 0.986 for quercetin and acteoside, respectively.

**Table 5:** Linear range, LOD, and LOQ parameters of quercetin and acteoside

Compound	Regression equation	R^2^	LOD (μg.ml^−1^)	LOQ (μg.ml^−1^)
Quercetin	y = 41041x + 1E + 06	0.999	3.82	12.73
Acteoside	y = 20899x + 2E + 06	0.986	2.8	8.6

*LOD* Limit of detection, *LOQ* limit of quantitation

## Conclusion

This study focused on the investigation of total phenolic and flavonoid contents, antioxidant, and cytotoxic activities of methanol extract and different fractions from *T. hyrcanicum* (family Lamiaceae). Among prepared samples, MF showed the highest amount of phenolic and flavonoid contents, and the best antioxidant activity. Also, MF significantly protected the NIH3T3 cell line against H_2_O_2_-induced oxidative stress by reducing MDA formation and inhibition of GSH oxidation. The results of the MTT assay demonstrated that EF had the highest cytotoxic activity. The phytochemical analysis resulted in the isolation and identification of two phenolic compounds (acteoside and quercetin). The isolated compounds had significant antioxidant activities and revealed protective effects on H_2_O_2_-induced oxidative stress, which was comparable with BHA (standard antioxidant). The acteoside had the best cytotoxicity against the A431 cancerous cell line. The HPLC quantification revealed that the methanol extract of this plant contained considerable amounts of acteoside and quercetin.

### Supplementary Information

**Additional file 1: Figure S1.** The separation elution curve for compound 1(acteoside): compound 1 was separated in Rt=22.4 min, using a semi-preparative HPLC. **Figure S2-1.** H-NMR spectra of compound 1 (acteoside). **Figure S2-2.** The expanded H-NMR spectra of compound 1 (acteoside). **Figure S2-3.** The expanded H-NMR spectra of compound 1 (acteoside). **Figure S3-1.** ^13^C-NMR spectra of compound 1 (acteoside). **Figure S3-2.** The expanded ^13^C-NMR spectra of compound 1 (acteoside). **Figure S3-3.** The expanded ^13^C-NMR spectra of compound 1 (acteoside). **Figure S3-4.** The expanded ^13^C-NMR spectra of compound 1 (acteoside). **Figure S3-5.** The expanded ^13^C-NMR spectra of compound 1 (acteoside). **Figure S4.** FT-IR spectra of compound 1 (acteoside). **Figure S5.** H-NMR spectra of compound 2 (quercetin). **Figure S6.** C-NMR spectra of compound 2 (quercetin). Figure S7: FT-IR spectra of compound 2 (quercetin).

## Data Availability

The datasets used and/or analyzed during the current study are available from the corresponding author on reasonable request.

## Abbreviations

BHA: Butylated hydroxyanisole
BHT: Butylated hydroxytoluene
ROS: Reacting oxygen species
DPPH: 2,2-Diphenyl-1-picrylhydrazyl
GA: Gallic acid
DMEM: Dulbecco's Modified Eagle Medium:
MTT: Methyl thiazol tetrazolium
ME: Methanol extract
EF: Ethyl acetate fraction
MF: Methanol fraction
HF: Hexane fraction
GAE: Gallic acid equivalent
QE: Quercetin equivalent
PRA: Phosphomolybdenum reduction assay
αTE: α-Tocopherol equivalent
TBA: Thiobarbituric acid
MDA: Malondialdehyde
GSH: Glutathione
DTNB: Dithionitrobenzoic acid
LOD: Limit of detection
LOQ: Limit of quantitation
