# Supplementary material for: Isolation and structure elucidation of the compounds from Teucrium hyrcanicum L. and the investigation of cytotoxicity, antioxidant activity, and protective effect on hydrogen peroxide-induced oxidative stress
Source: BMC Complement Med Ther. 2023 Dec 12;23:447. doi: 10.1186/s12906-023-04262-8 (PMC10714485; doi:10.1186/s12906-023-04262-8)
Supplement: Supplementary file 1 — Additional file 1: Figure S1. The separation elution curve for compound 1(acteoside): compound 1 was separated in Rt=22.4 min, using a semi-preparative HPLC. Figure S2-1. H-NMR spectra of compound 1 (acteoside). Figure S2-2. The expanded H-NMR spectra of compound 1 (acteoside). Figure S2-3. The expanded H-NMR spectra of compound 1 (acteoside). Figure S3-1. 13C-NMR spectra of compound 1 (acteoside). Figure S3-2. The expanded 13C-NMR spectra of compound 1 (acteoside). Figure S3-3. The expanded 13C-NMR spectra of compound 1 (acteoside). Figure S3-4. The expanded 13C-NMR spectra of compound 1 (acteoside). Figure S3-5. The expanded 13C-NMR spectra of compound 1 (acteoside). Figure S4. FT-IR spectra of compound 1 (acteoside). Figure S5. H-NMR spectra of compound 2 (quercetin). Figure S6. C-NMR spectra of compound 2 (quercetin). Figure S7: FT-IR spectra of compound 2 (quercetin). [file 12906_2023_4262_MOESM1_ESM.docx]

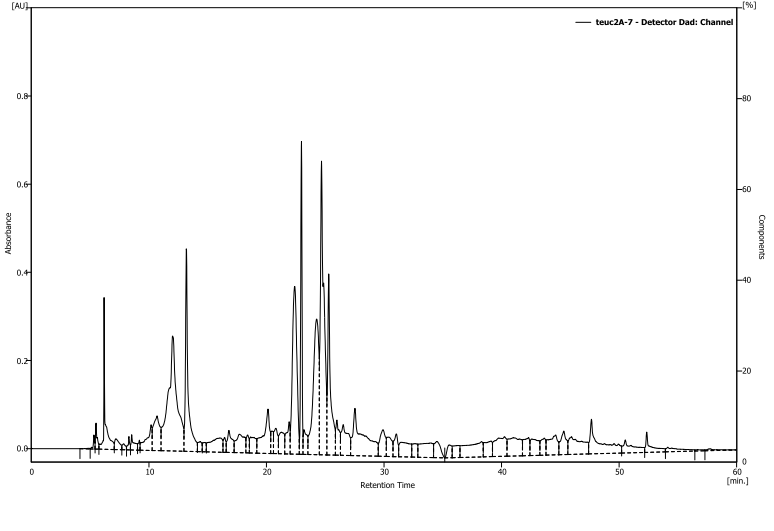


Figure S1: The separation elution curve for compound 1(acteoside): compound 1 was separated in Rt=22.4 min, using a semi-preparative HPLC


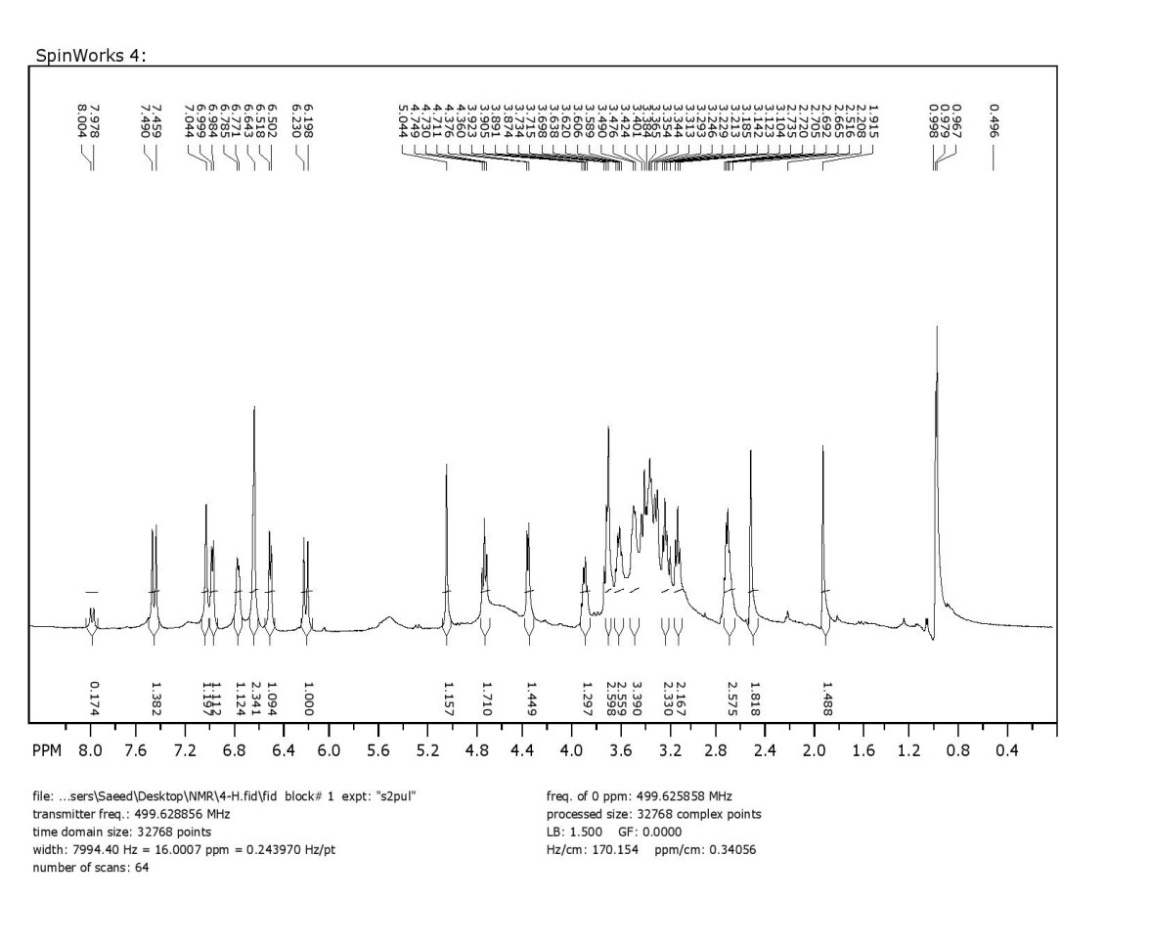


Figure S2-1: H-NMR spectra of compound 1 (acteoside)


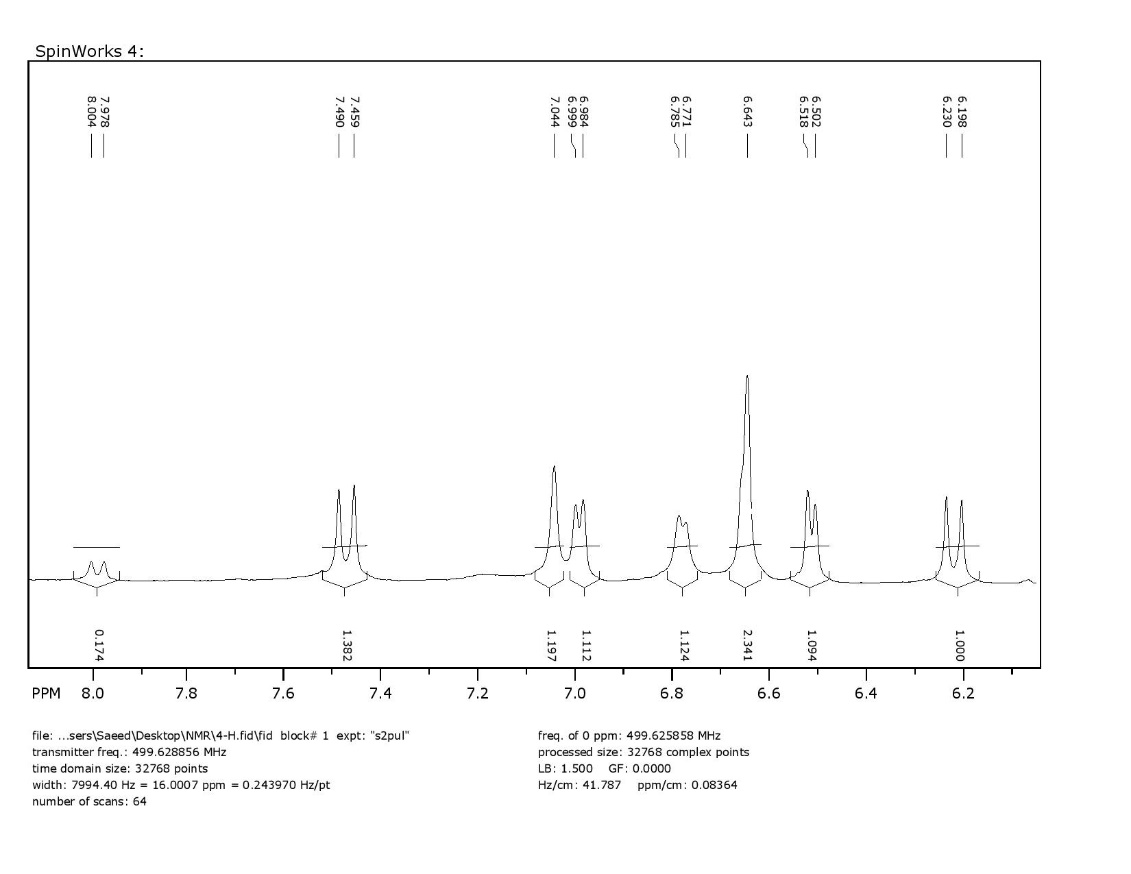


Figure S2-2: The expanded H-NMR spectra of compound 1 (acteoside)


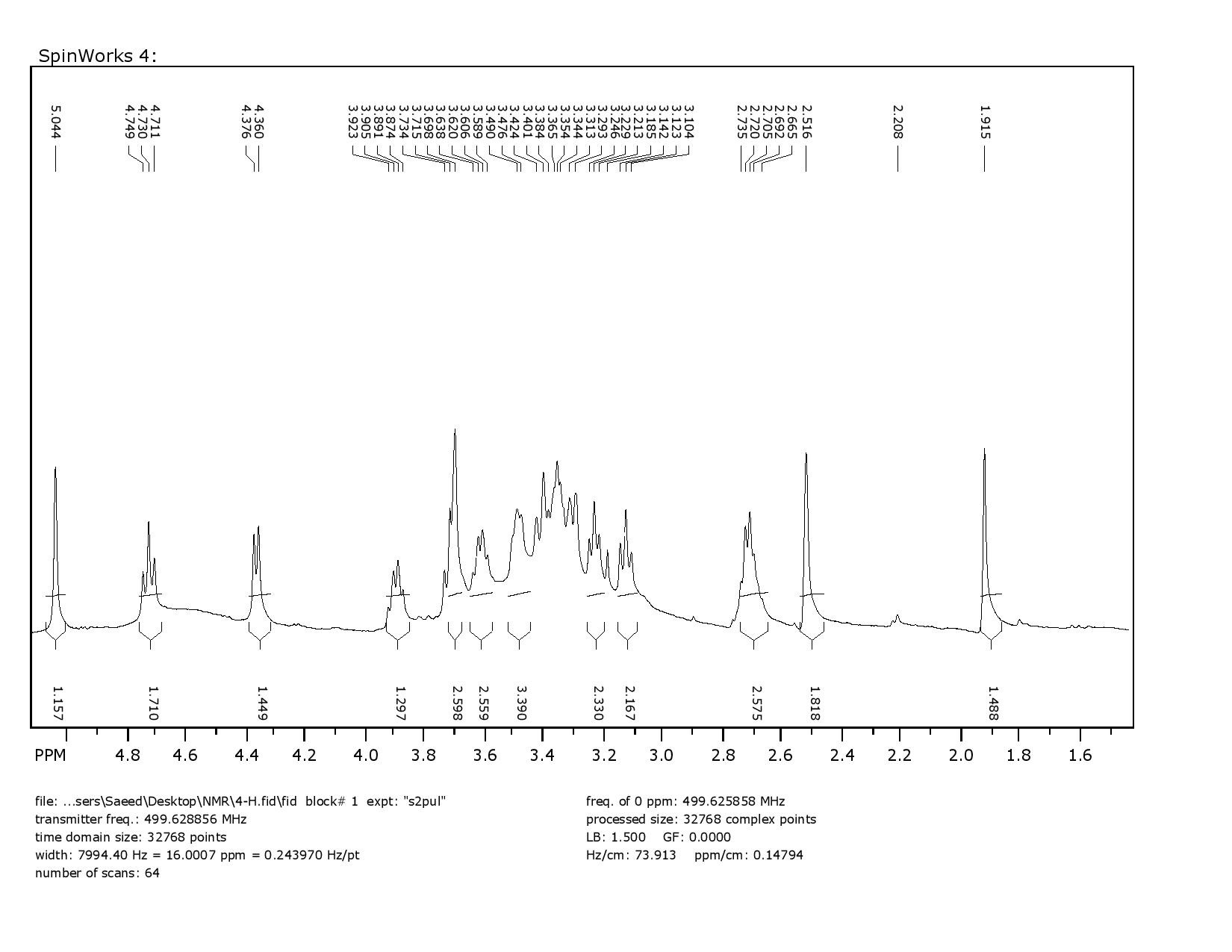


Figure S2-3: The expanded H-NMR spectra of compound 1 (acteoside)


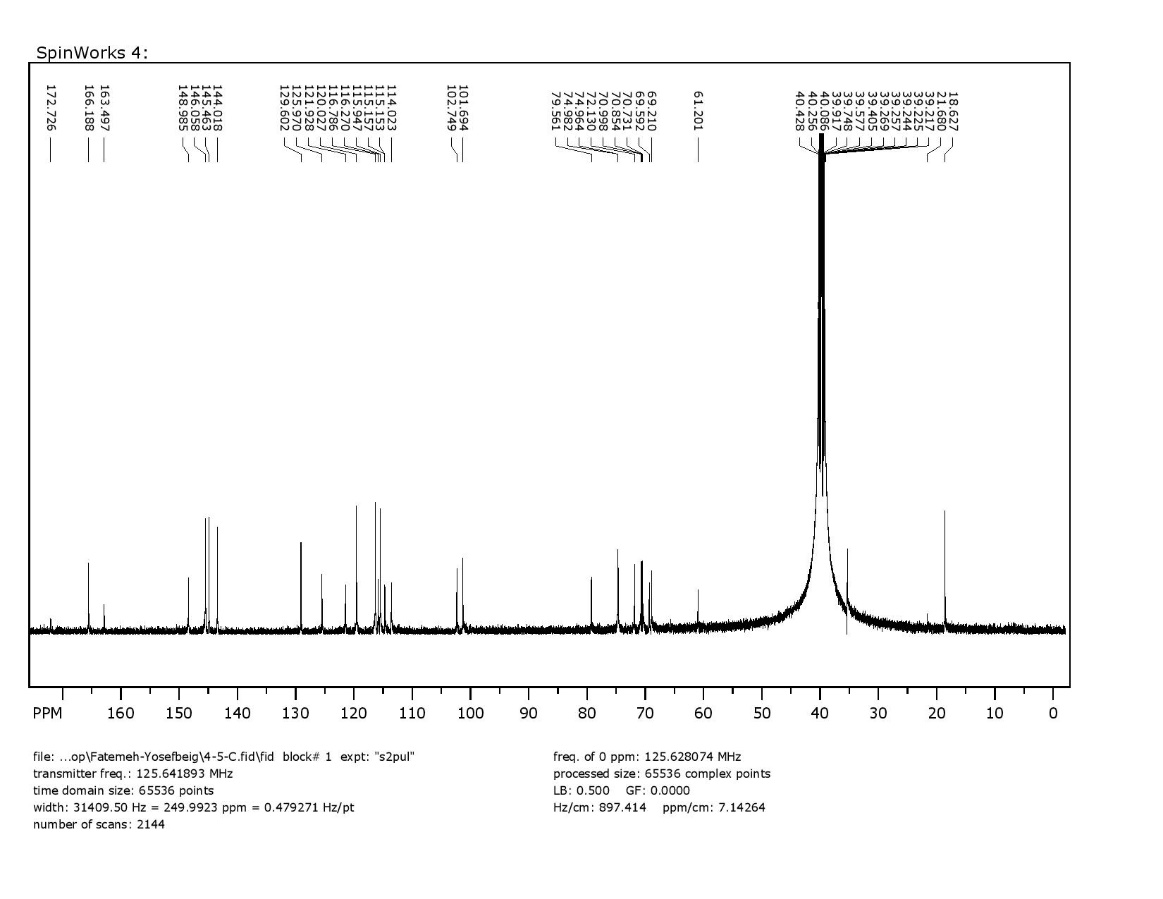


Figure S3-1:^13^C-NMR spectra of compound 1 (acteoside)


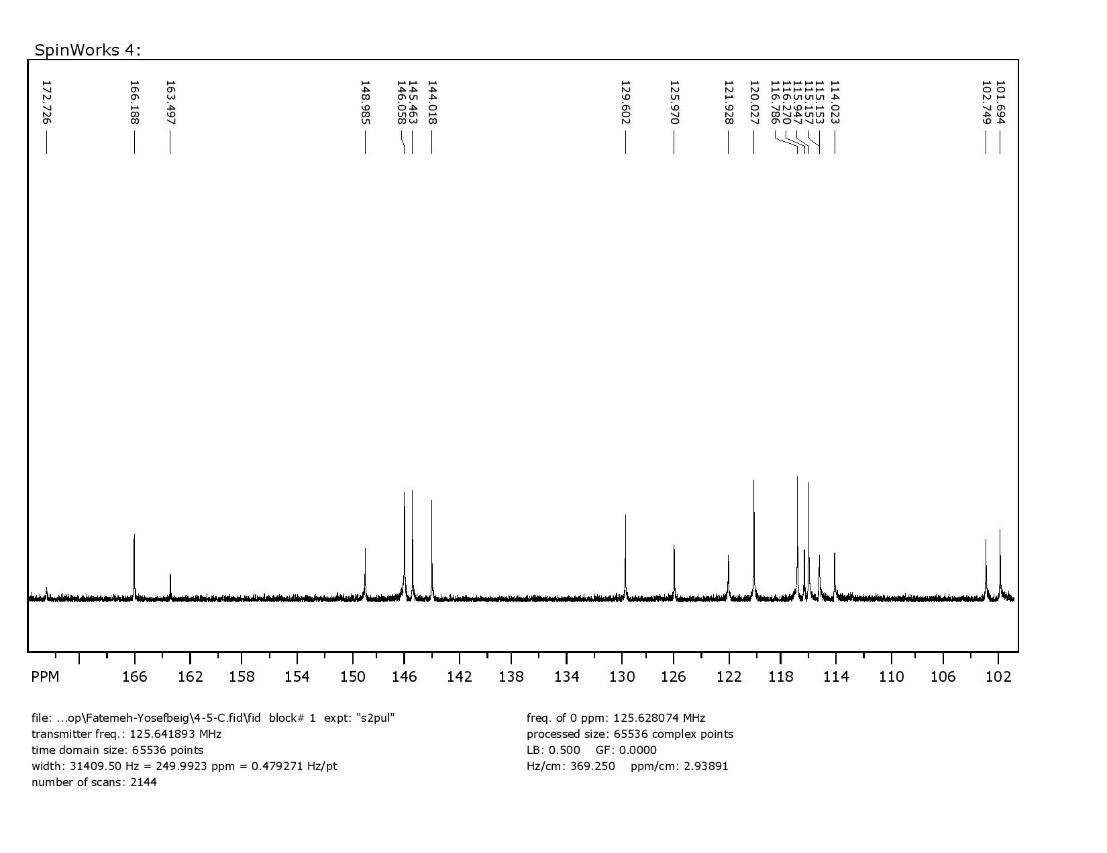


Figure S3-2: The expanded ^13^C-NMR spectra of compound 1 (acteoside)


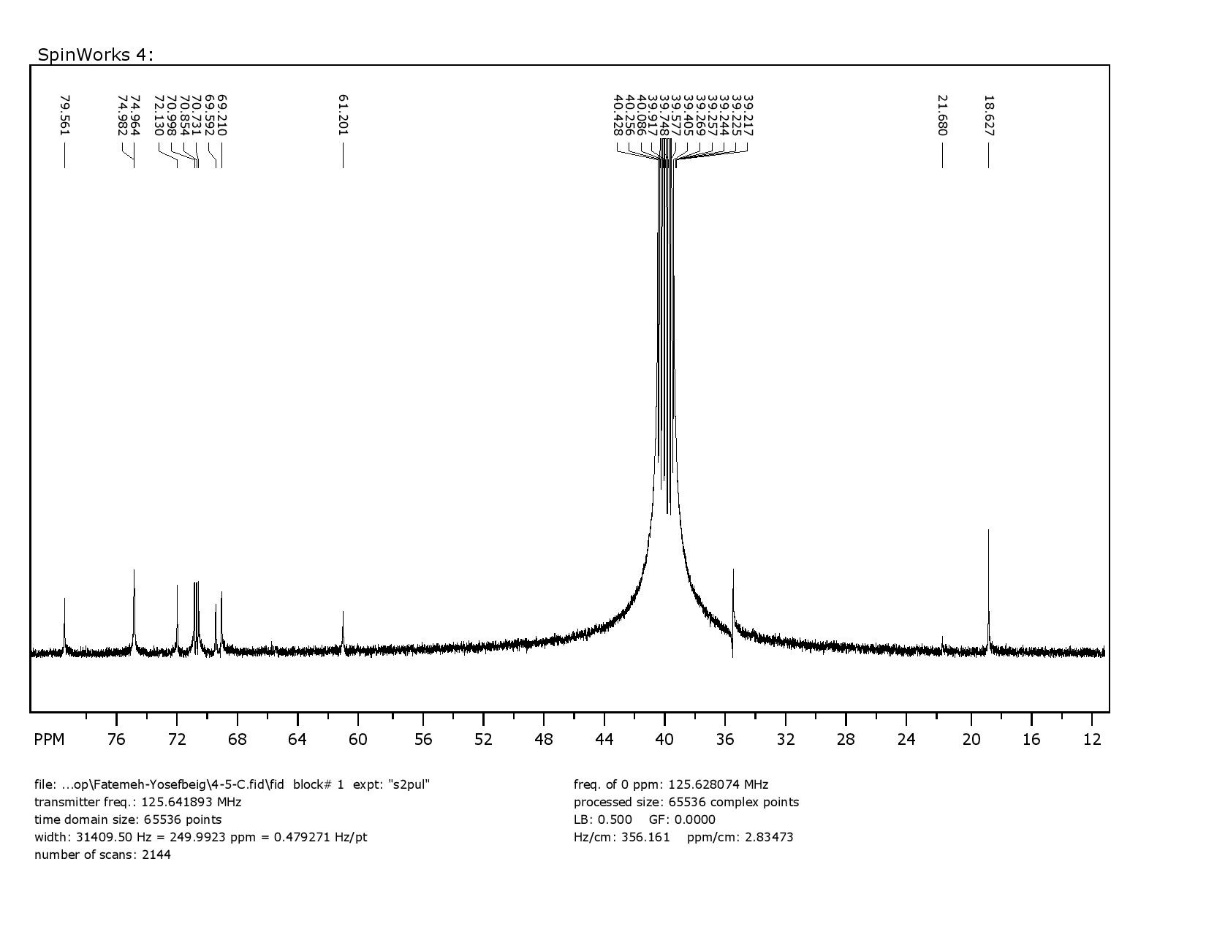


Figure S3-3: The expanded ^13^C-NMR spectra of compound 1 (acteoside)


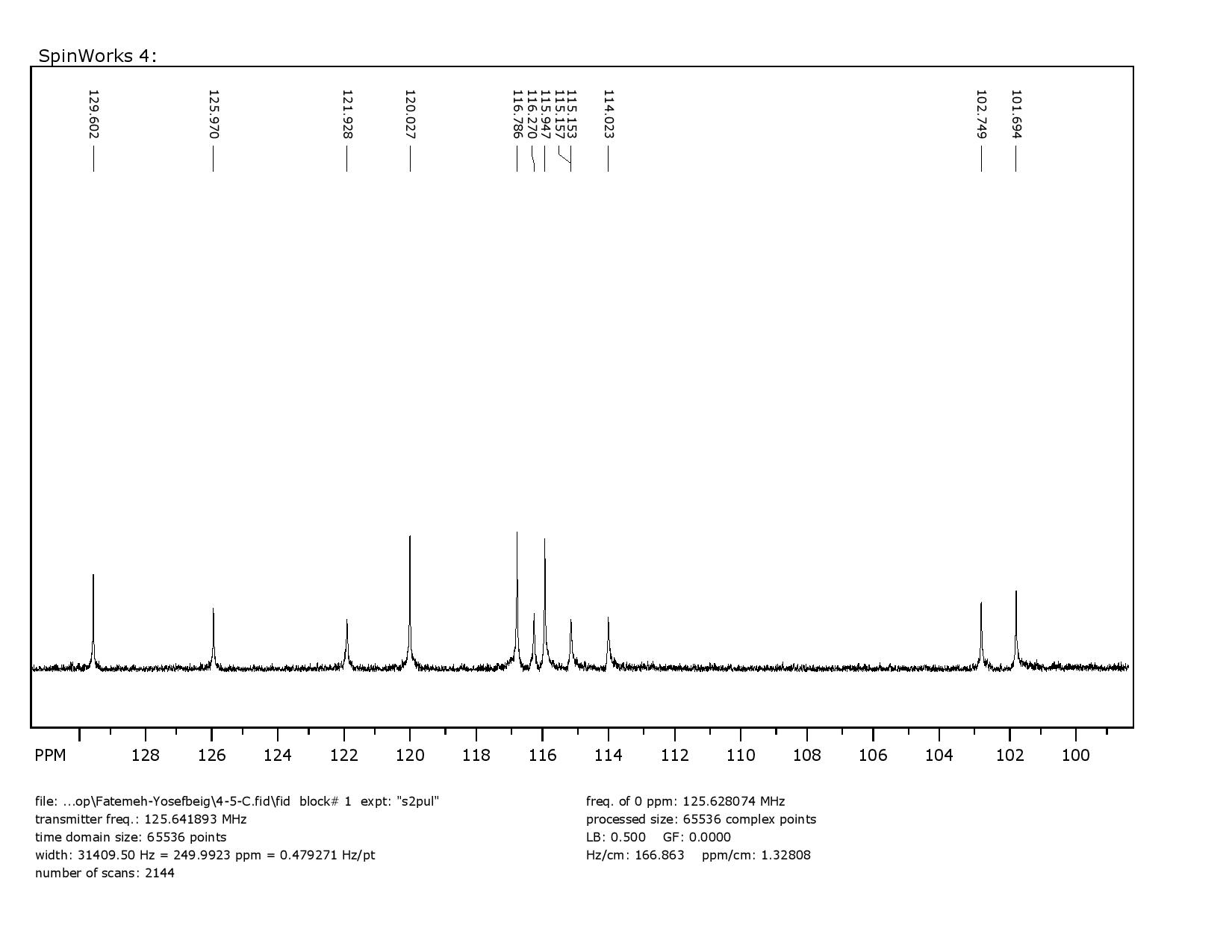


Figure S3-4: The expanded ^13^C-NMR spectra of compound 1 (acteoside)


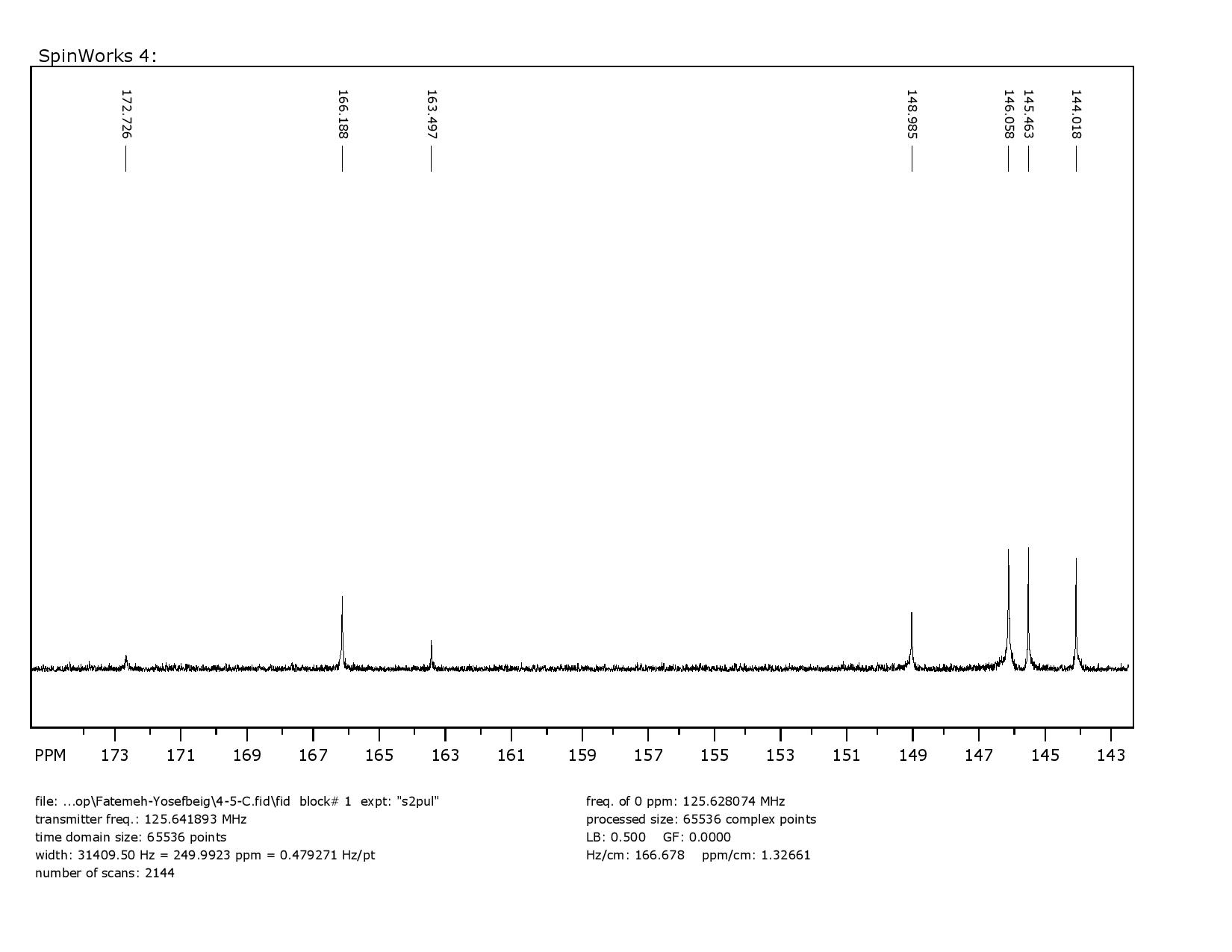


Figure S3-5: The expanded ^13^C-NMR spectra of compound 1 (acteoside)


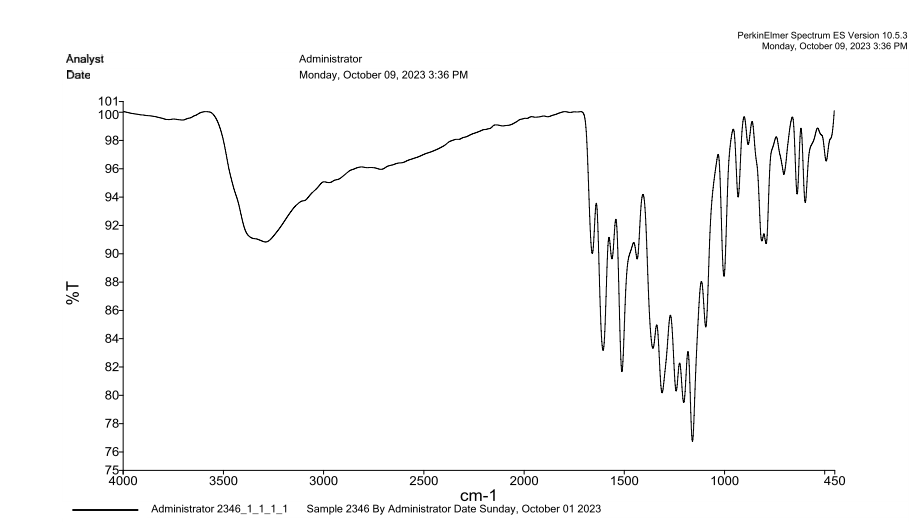


Figure S4: FT-IR spectra of compound 1 (acteoside)


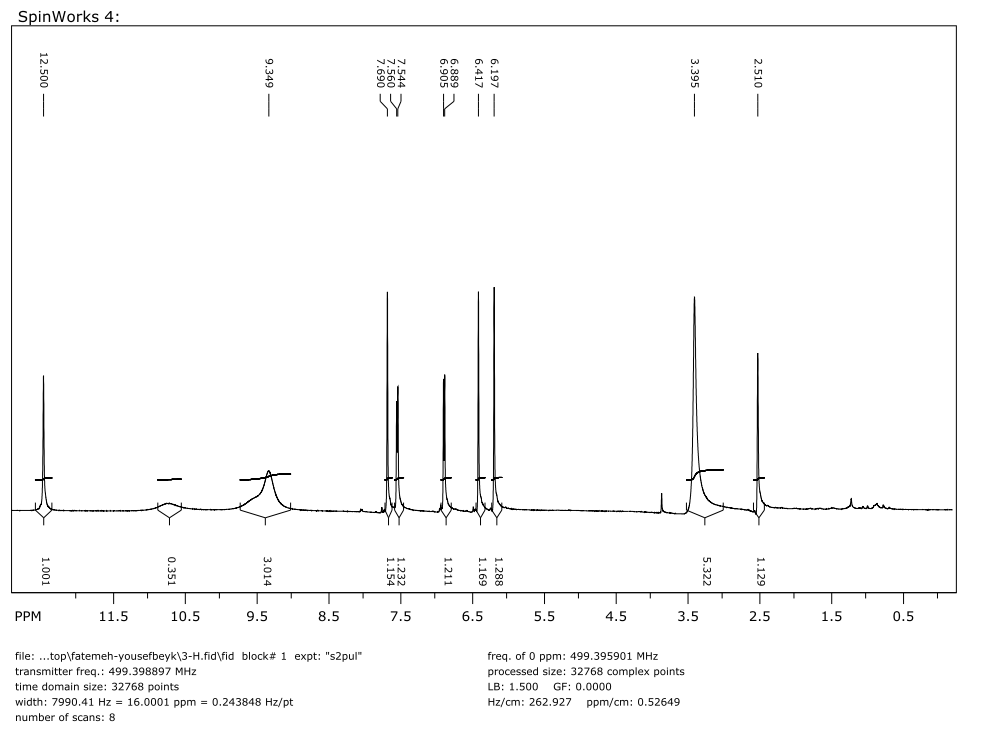


Figure S5: H-NMR spectra of compound 2 (quercetin)


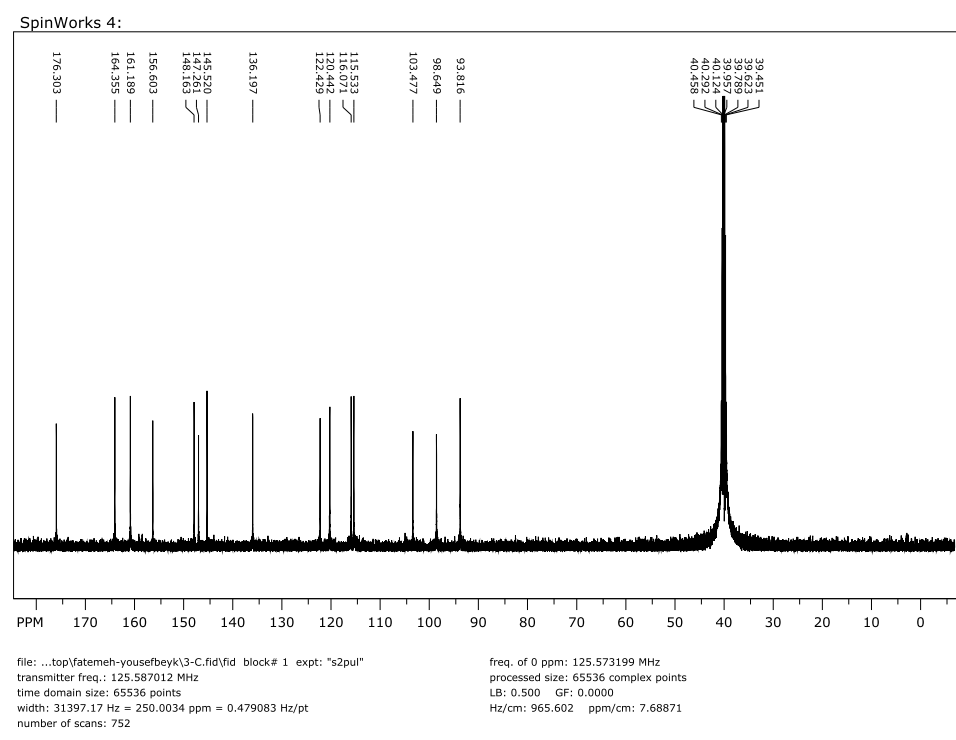


Figure S6: C-NMR spectra of compound 2 (quercetin)


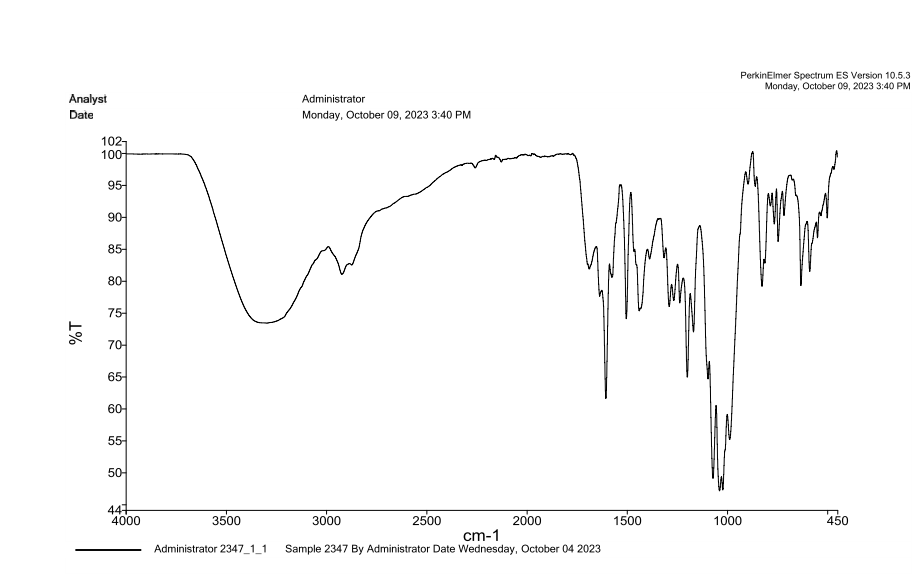


Figure S7: FT-IR spectra of compound 2 (quercetin)
